# RNA Misprocessing in *C9orf72*-Linked Neurodegeneration

**DOI:** 10.3389/fncel.2017.00195

**Published:** 2017-07-11

**Authors:** Holly V. Barker, Michael Niblock, Youn-Bok Lee, Christopher E. Shaw, Jean-Marc Gallo

**Affiliations:** Department of Basic and Clinical Neuroscience, Maurice Wohl Clinical Neuroscience Institute, Institute of Psychiatry, Psychology and Neuroscience, King’s College London London, United Kingdom

**Keywords:** *C9orf72*, amyotrophic lateral sclerosis, frontotemporal dementia, RNA, repeats, splicing

## Abstract

A large GGGGCC hexanucleotide repeat expansion in the first intron or promoter region of the *C9orf72* gene is the most common genetic cause of familial and sporadic Amyotrophic lateral sclerosis (ALS), a devastating degenerative disease of motor neurons, and of Frontotemporal Dementia (FTD), the second most common form of presenile dementia after Alzheimer’s disease. *C9orf72*-associated ALS/FTD is a multifaceted disease both in terms of its clinical presentation and the misregulated cellular pathways contributing to disease progression. Among the numerous pathways misregulated in *C9orf72*-associated ALS/FTD, altered RNA processing has consistently appeared at the forefront of *C9orf72* research. This includes bidirectional transcription of the repeat sequence, accumulation of repeat RNA into nuclear foci sequestering specific RNA-binding proteins (RBPs) and translation of RNA repeats into dipeptide repeat proteins (DPRs) by repeat-associated non-AUG (RAN)-initiated translation. Over the past few years the true extent of RNA misprocessing in *C9orf72*-associated ALS/FTD has begun to emerge and disruptions have been identified in almost all aspects of the life of an RNA molecule, including release from RNA polymerase II, translation in the cytoplasm and degradation. Furthermore, several alterations have been identified in the processing of the *C9orf72* RNA itself, in terms of its transcription, splicing and localization. This review article aims to consolidate our current knowledge on the consequence of the *C9orf72* repeat expansion on RNA processing and draws attention to the mechanisms by which several aspects of *C9orf72* molecular pathology converge to perturb every stage of RNA metabolism.

## Introduction

Amyotrophic lateral sclerosis (ALS) is a fatal progressive neuromuscular disease resulting from the degeneration of motor neurons in the brain and spinal cord for which no effective treatments are currently available. Frontotemporal Dementia (FTD), the second most common form of dementia after Alzheimer’s disease, is characterized by behavioral and language deficits and manifests pathologically by neuronal atrophy in the frontal and anterior temporal lobes in the brain. A hexanucleotide G_4_C_2_ repeat expansion in the first intron or promoter region of the *C9orf72* gene on chromosome 9p21 represents the most common familial cause of ALS and FTD (Dejesus-Hernandez et al., 2011; Renton et al., 2011). C9orf72 repeat expansions manifest pathologically as proteinaceous inclusions of the RNA/DNA binding protein TDP-43. *C9orf72* repeat expansion as a common genetic cause of ALS of FTD further emphasizes the extensive clinical, genetic and pathological overlap between these two conditions, suggesting that both diseases represent opposite ends of a continuous clinical spectrum. *C9orf72* lies at the heart of this spectrum, with the risk of developing FTD and ALS concurrently increasing from 7% to 30% in expansion carriers (van der Zee et al., 2013). The number of repeats in the normal population ranges from 2 to 23. Expansion sizes vary considerably between individual cases and expansion sizes as high as 3500 repeats have been reported (Chen et al., 2016), however the lower threshold required to initiate pathogenesis is poorly defined. A report described an individual with an intermediate repeat length (30 units) exhibiting some *C9orf72*-associated pathological phenotypes in the absence of clinical manifestations (Gami et al., 2015) suggesting that 30 repeats may lie on the border between initial cellular abnormalities and full-blown disease. Expansions of less than 30 repeats are not typically associated with disease; however a minority of ALS cases with 20–22 repeats have been described (Byrne et al., 2014). Furthermore, such intermediate repeat sizes significantly increase the risk of developing ALS (Chen et al., 2016) and are associated with decreased *C9orf72* promoter activity (Gijselinck et al., 2016).

**Figure 1:**
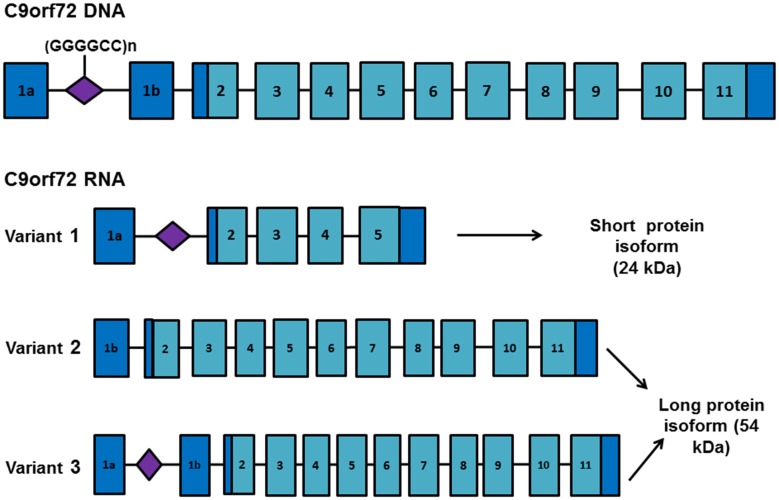
*C9orf72* RNA transcript variants. Schematic representation of the *C9orf72* gene and RNA transcript variants. Exons are depicted as blue boxes and the location of the GGGGCC repeat expansion is shown in purple. Differential selection of transcription start and termination sites generates three different RNA transcripts. Variant 1 encodes a short protein isoform (isoform B) whereas variants 2 and 3 encode a longer protein isoform (isoform A). Presence of the repeat expansion favors transcription from exon 1a, increasing the proportion of transcripts containing the repeat expansion.

Differential use of transcription alternative start and termination sites generates three RNA transcripts from *C9orf72* DNA. These encode two protein isoforms consisting of a long isoform (isoform A) of approximately 54 kDa derived from variants 2 (NM_018325.4) and 3 (NM_001256054.2), and a short isoform (isoform B) of approximately 24 kDa derived from variant 1 (NM_145005.6; Figure 1). Several studies have shown a reduction in *C9orf72* mRNA (Dejesus-Hernandez et al., 2011; Renton et al., 2011; Gijselinck et al., 2012) and protein levels (Waite et al., 2014) in expansion carriers, suggesting that toxicity may be mediated by loss of function of the *C9orf72*-encoded protein. However, neural-specific ablation of *C9orf72* or knockdown using antisense oligonucleotides (ASOs) in mice has failed to recapitulate pathology (Lagier-Tourenne et al., 2013; Koppers et al., 2015; Atanasio et al., 2016; Jiang et al., 2016; O’Rourke et al., 2016). *C9orf72* knockdown in murine models produces an altered immune response (Atanasio et al., 2016) characterized by the accumulation of lysosomal vesicles within macrophages, implicating a role for the C9orf72 protein in the regulation of late endosomal/lysosomal trafficking in macrophages and microglia (O’Rourke et al., 2016). The *C9orf72* protein functions as a guanine exchange factor (GEF) for Rab GTPases to regulate vesicular trafficking and autophagy (Webster et al., 2016). Several independent reports have shown that the *C9orf72* long isoform forms a complex with WDR41 and SMCR8 proteins, both regulators of autophagy (Blokhuis et al., 2016; Sellier et al., 2016; Sullivan et al., 2016; Xiao et al., 2016).

**Figure 2:**
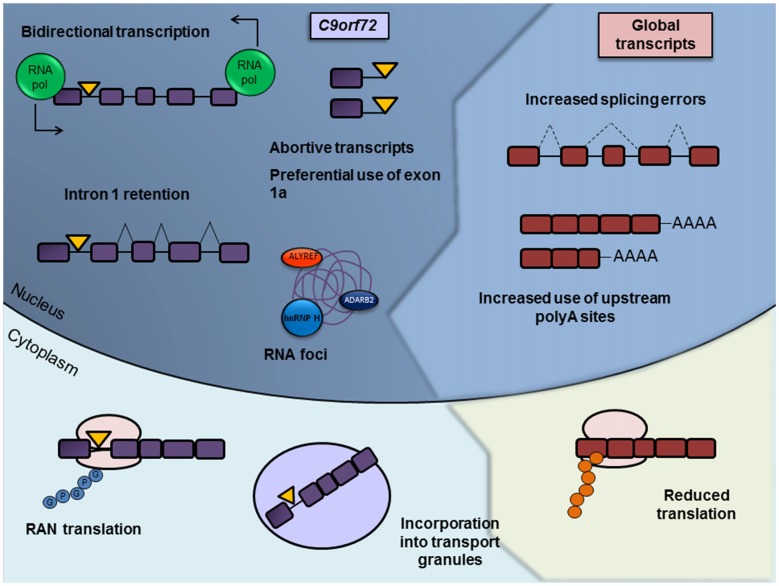
Summary of misregulated RNA processing events in c9ALS/FTD. RNA processing abnormalities in c9ALS/FTD brain tissue or in cellular and animal models have been defined in almost every aspect of RNA processing, from DNA transcription to its translation and eventual degradation. Misregulated and/or pathogenic processes involving the expanded *C9orf72* transcript itself are depicted on the left hand side and labeled in purple, with the repeat expansion represented as a yellow triangle. Such processes include bidirectional transcription of the repeat-containing allele, abortive transcription, decreased splicing of the repeat-containing intron, sequestration of RNA binding proteins by RNA foci, repeat-associated non-AUG (RAN) translation and the incorporation of the expanded transcript into RNA transport granules. Disrupted general RNA processing pathways are shown on the right and labeled in red, including decreased splicing consistency, differential use of polyadenylation sites (PASs) and reductions in translation.

It is becoming increasingly apparent that altered RNA processing plays a key role in *C9orf72*-mediated toxicity through two separate, albeit related, aspects of RNA processing (Figure 2). The first is altered processing of the expanded *C9orf72* transcript itself, in terms of altered transcription, splicing defects, nuclear aggregation and non-conventional translation. The second represents downstream and indirect changes in RNA processing of other transcripts. Far from being disparate entities in their role in *C9orf72* toxicity, we highlight how RNA and protein frequently interact and drive disturbances in RNA metabolism. In this review article we describe aspects of the pathomechanism of *C9orf72*-associated ALS/FTD linked to disturbances at the RNA level and how these changes may drive further RNA processing abnormalities.

## TDP-43 Is an Essential Mediator of RNA Metabolism

A characteristic pathological hallmark of ~90% of ALS and ~60% of FTD cases, including c9ALS/FTD cases is the presence of cytoplasmic, ubiquitin-positive, inclusions of the DNA/RNA binding protein, TDP-43, in affected neurons (Neumann et al., 2006; Mackenzie et al., 2014). In disease, TDP-43 aggregates in the cytoplasm where it undergoes a series of post-translational modifications, including ubiquitination, phosphorylation and C-terminal cleavage (Neumann et al., 2006). TDP-43 is mainly a nuclear protein and its mislocalization to the cytoplasm and aggregation are associated with a reduction and subsequent loss of function. Mislocalization of TDP-43 from the nucleus to the cytoplasm is likely to be a key mediator of pathogenesis, as mutations in *TARDBP*, the gene encoding TDP-43, are causative of some forms of familial ALS (Kabashi et al., 2008; Sreedharan et al., 2008).

TDP-43 is a highly conserved RNA-binding protein (RBP), possessing two RNA recognition motif (RRM) domains. TDP-43 regulates splicing, RNA turnover and transport and therefore interacts with a myriad of proteins involved in RNA metabolism (Buratti and Baralle, 2010; Blokhuis et al., 2016). Downregulation of TDP-43 expression in the CNS alters the expression of ~600 transcripts and splicing of over 900 genes, the majority of which are implicated in synaptic transmission, neuronal development and RNA metabolism (Polymenidou et al., 2011; Tollervey et al., 2011). Depletion of TDP-43 reduces the expression of long intron-containing transcripts, the majority of these essential for maintaining neuronal integrity (Lagier-Tourenne et al., 2012). ALS-causing TDP-43 mutations can also disrupt the splicing pattern of its mRNA targets in the absence of TDP-43 mislocalization and aggregation, resulting in abnormal splicing of transcripts involved in neurological function (Arnold et al., 2013). One function of TDP-43 is that it serves to repress splicing of nonconserved cryptic exons (Ling et al., 2015). Cryptic exons are stretches of nucleotides surrounded by sequences similar to authentic splice sites but which are not normally recognized as such so are not spliced into the wild-type RNA. Inclusion of a cryptic exon often results in the introduction of a premature stop codon, resulting in either protein truncation or mRNA degradation through nonsense-mediated decay. Cryptic exon inclusion occurs in Alzheimer’s brain exhibiting TDP-43 nuclear clearance in the absence of inclusion formation, suggesting that cryptic exon inclusion occurs as an early event among TDP-43 proteinopathies (Sun et al., 2017).

TDP-43 not only serves to regulate coding RNAs but is also implicated in the regulation of non-coding RNA expression, including microRNA (miRNA) and long noncoding RNA (lncRNA). TDP-43 associates with both microprocessor complexes and its depletion results in reduced binding efficiency of both complexes to a subset of miRNAs (Kawahara and Mieda-Sato, 2012). In addition to facilitating miRNA biogenesis on a global level, TDP-43 can directly bind miRNAs or their precursors, including let-7b, miR-663 (Buratti et al., 2010) and miR-9 (Zhang et al., 2013). TDP-43 depletion results in downregulation of let-7b which in turn is associated with altered expression levels of several let-7b targets. (Buratti et al., 2010). Included in these targets are STX3 and VAMP3 which are involved in synaptic vesicle formation and synaptic exocytosis, respectively (Darios and Davletov, 2006; Deák et al., 2006). Another transcript affected by let-7b downregulation is DYRK1A, upregulation of which is associated with neuronal deficits in individuals with Downs syndrome (Lepagnol-Bestel et al., 2009).

LncRNAs make up an extensive family of long (several kb) RNA molecules which serve key regulatory roles and have been implicated in numerous cellular pathways. In cases of sporadic frontotemporal lobar degeneration with TDP-43 pathology, TDP-43 exhibits significantly increased binding to *MALAT* and *NEAT1* lncRNAs (Tollervey et al., 2011). *MALAT1* influences the phosphorylation patterns of the SR-family of splicing proteins and recruits splicing factors to nuclear speckles (Tripathi et al., 2010). *NEAT1* is responsible for paraspeckle assembly (Clemson et al., 2009), nuclear domains implicated in transcriptional regulation, pre-mRNA splicing and mRNA nuclear retention. Downregulation of TDP-43 results in a corresponding decrease in *MALAT1* expression (Guo et al., 2015), suggesting that loss of TDP-43 nuclear function could result in splicing abnormalities through altered expression of *MALAT1* lncRNA.

Furthermore, TDP-43 facilitates transport and local translation of its mRNA targets within distal neuronal regions. In *Drosophila* motor neurons, mouse cortical neurons and motor neurons derived from human induced pluripotent stem cells (iPSCs), TDP-43 associates with bidirectionally transported messenger ribonucleoprotein complex (mRNP) granules. In neuronal cells harboring ALS-causing TDP-43 mutations, the anterograde transport of mRNP granules is selectively impaired (Alami et al., 2014), suggesting that impaired axonal transport of TDP-43 target mRNAs may contribute to ALS-associated pathogenesis.

Due to the multitudinous roles of TDP-43 in the metabolism of not only messenger RNAs, but also miRNAs and lncRNAs, it is perhaps unsurprising that TDP-43 proteinopathies present with disruptions in RNA at so many levels. Defective RNA processing may represent a common pathway connecting FTD and ALS, although the mechanism by which TDP-43 nuclear clearance and aggregation results in such clinical heterogeneity remains ambiguous.

## RNA Toxicity: The Role of RNA-Binding Protein Sequestration in Altered RNA Processing Events

*C9orf72* repeat RNA is bidirectionally transcribed, from both the sense (G_4_C_2_)_n_ and the antisense (G_2_C_4_)_n_ DNA strand (Zu et al., 2013), a phenomenon which appears to be more widespread than previously recognized (Pelechano and Steinmetz, 2013). Repeat RNA forms punctate nuclear aggregates termed RNA foci. Multiple nuclear, and more rarely, cytoplasmic, sense and antisense RNA foci have been identified in patient-derived cells and throughout the CNS in expansion carriers (Gendron et al., 2013; Lagier-Tourenne et al., 2013; Mizielinska et al., 2013; Zu et al., 2013). Analyses of the distribution and abundance of RNA foci initially revealed a significant correlation with clinical phenotype (Cooper-Knock et al., 2014) and an inverse correlation with age of onset (Mizielinska et al., 2013), implicating RNA foci formation as a key driver of *C9orf72* toxicity. However, extensive characterization in a larger cohort found no association between RNA foci distribution and clinical phenotype. RNA foci load is highest in cerebellar Purkinje cells, the loss of which has not been described in *C9orf72* expansion carriers. Interestingly, a higher percentage of antisense foci in the frontal cortex appear to correlate with a later age of onset, particularly in patients diagnosed with FTLD (DeJesus-Hernandez et al., 2017).

Since the discovery of RNA foci in c9ALS/FTD patient tissue, RNA toxicity has emerged as a leading hypothesis for *C9orf72*-associated pathogenesis. The RNA toxicity hypothesis postulates that G_4_C_2_ repeats accumulate into nuclear RNA foci and sequester essential RBPs, impairing their ability to regulate their RNA targets and culminating in a range of RNA misprocessing events. RNA pull-down and colocalization experiments in c9ALS brain and neurons derived from iPSCs (iPSNs) have identified a panel of RBPs binding to G_4_C_2_ repeat RNA, including hnRNP A1, hnRNP A3, hnRNP H, ADARB2, Pur-α, ASF/SF2, ALYREF and nucleolin (Donnelly et al., 2013; Lee et al., 2013; Mori et al., 2013a; Sareen et al., 2013; Xu et al., 2013; Cooper-Knock et al., 2014; Haeusler et al., 2014). Sense and antisense RNA foci sequester a similar panel of RBPs, specifically SC35, hnRNP K, hnRNP A1, ALYREF and hnRNP H (Cooper-Knock et al., 2014). Interestingly, only antisense foci demonstrate a significant positive correlation with TDP-43 mislocalization in motor neurons of c9ALS patients (Cooper-Knock et al., 2015b), which in turn is associated with neurodegeneration (Davidson et al., 2016). ASOs targeting the *C9orf72* transcript downstream of the repeats reduce RNA foci levels (Donnelly et al., 2013; Jiang et al., 2016), attenuate sequestration of specific RBPs and normalize gene expression changes (Donnelly et al., 2013), suggesting that transcriptome alterations represent a direct downstream consequence of G_4_C_2_ RNA toxicity. Upregulation of *C9orf72* mRNA expression is associated with concomitant downregulation of genes enriched for functions in RNA metabolism, such as genes encoding tRNA synthetases (Nataf and Pays, 2015) emphasizing the role of tRNA metabolism in motor neuron degeneration (Weitzer et al., 2015). Such downregulation of genes involved in the regulation of RNA metabolism is consistent with RBP sequestration by the *C9orf72* hexanucleotide repeat expansion, as the reduced pool of RBPs is less able to promote the expression of such transcripts.

## DPR Accumulation Is Detrimental to Numerous Cellular Pathways

Sense and antisense *C9orf72* RNA repeats are translated by an unconventional form of translation, repeat-associated non-AUG initiated translation (RAN translation) (Box 1). RAN translation was first described in the context of spinocerebellar ataxia type 8 (SCA8) caused by a CTG repeat expansion. RAN translation of (G_4_C_2_)_._(C_4_G_2_)_n_ results in the synthesis of five dipeptide repeat protein (DPR) species; poly-GA, poly-GP, poly-GR, from the sense transcript and poly-GP, poly-PR and poly-PA, from the antisense transcript. All five DPR species form ubiquitinated inclusions in cerebellum, hippocampus and other brain regions in expansion carriers (Ash et al., 2013; Gendron et al., 2013; Mann et al., 2013). Several studies have demonstrated the toxicity of DPRs *in vitro* and *in vivo* (Kwon et al., 2014; Mizielinska et al., 2014; Wen et al., 2014; Zhang et al., 2014). Poly-GA expression in primary neurons reduces dendritic branching, increases apoptosis through caspase-3 activation (May et al., 2014) and induces ER stress (Zhang et al., 2014). The arginine-rich DPRs, poly-GR and poly-PR, exhibit the most robust toxicity in transgenic fly models and motor neuron cultures (Mizielinska et al., 2014; Wen et al., 2014). Expression of (PR)_50_ and (GR)_50_ dramatically decreases survival of primary cortical and motor neurons, presumably through a pathway of translational dysregulation (Wen et al., 2014). Poly-PR and poly-GR irreversibly bind nucleoli and can alter the splicing patterns of specific RNAs. For instance, when applied to cultured human astrocytes (PR)_20_ penetrates cells and causes exon-skipping in *RAN* and *PTX3* RNA, resulting in the production of mRNAs encoding truncated proteins and in impaired biogenesis of ribosomal RNA, suggestive of nucleolar dysfunction (Kwon et al., 2014). A yeast genetic screen demonstrated that deletion of specific genes involved in ribosomal RNA processing, such as *NOB1* and *NSR1*, suppressed poly-PR toxicity (Jovičić et al., 2015). Consistent with these findings, a separate study identified nuclear inclusions of poly-PR and poly-GR tightly bound to nucleolar proteins, resulting in enlargement of the nucleus and induction of apoptosis (Wen et al., 2014).

Box 1 **The mechanism of repeat-associated non-AUG (RAN) translation.**

The type of repeat-containing RNA species that are exported to the cytoplasm and subject to RAN translation is still to be elucidated. However several recent results support the notion that the substrate for RAN translation of G_4_C_2_ repeats into dipeptide repeat proteins (DPRs) is a mature mRNA. First G_4_C_2_ repeats have been shown to cause overt neurotoxicity in *Drosophila* only if there are in the context of an mRNA (Tran et al., 2015). Second, initiation of RAN translation at CGG repeats, causing Fragile X tremor ataxia syndrome (FXTAS), requires a 5′ 7-methylguanosine (m^7^G) cap (Kearse et al., 2016). Addition of an m^7^G cap is part of the final processing of mRNA. In patient tissues, the repeat-containing intron has been shown to be retained in a proportion of polyadenylated *C9orf72* RNA species in which downstream exons were spliced correctly resulting in a *C9orf72* mRNA with an enlarged 5′-untranslated region (5′-UTR) containing the G_4_C_2_ repeat domain (Niblock et al., 2016). These species would be exported to the cytoplasm through the conventional pathway of mRNA export and have a 5′ m^7^G cap and are therefore the prime candidates for the template for RAN translation of G_4_C_2_ repeats into DPRs.

Finally, the study of Kearse et al. (2016) revealed that the mechanism of RAN translation may more closely resemble the canonical mode of translation than previously thought. The canonical mode of translation initiation occurs by recruitment of various initiation factors to the 5′ m^7^G cap, which scan the mRNA in search of an AUG initiation codon. In the case of microsatellite repeat disorders in which translation occurs in the absence of an AUG codon, RAN translation may occur in a similar fashion to internal ribosomal entry site (IRES)-mediated translation initiation (Chiang et al., 2001), in which secondary structures directly recruit initiation factors. Indeed, previous studies have identified a requirement for the formation of hairpin secondary structures to initiate RAN translation, as repeat sequences unable to form such secondary structures are not subject to RAN translation (Zu et al., 2011). However, RAN translation of CGG repeats is dependent on the m^7^G cap and eIF4E and eIF4A initiation factors, utilizing a scanning mechanism to initiate translation at codons which slightly deviate from the AUG sequence, located upstream of the repeats or within the repeat sequence itself. The secondary structure formed by CGG repeats may stall the initiation complex as it scans the mRNA transcript, resulting in the utilization of near-AUG sequences upstream of the repeats as alternative initiation codons (Kearse et al., 2016).

While analyses of DPR interactomes have revealed preferential interactions between poly-GA and proteins involved in proteasomal degradation (May et al., 2014; Zhang et al., 2016), the arginine-rich DPRs bind to numerous interactors enriched in RBPs and components of membrane-less organelles (Lee et al., 2016; Lin et al., 2016; Boeynaems et al., 2017). A significant number of these proteins possess low complexity sequence domains (LCDs) which mediate the assembly of membrane-less organelles such as RNA granules, nucleoli, spliceosomes and the nuclear pore complex (NPC). In order for membrane-less organelles to form they must separate from the liquid cytoplasm, which is achieved by concentrating the organelle components and the formation of a network of weak multi-valent interactions, a process known as liquid-liquid phase separation (LLPS) (Hyman et al., 2014). hnRNPs including FUS, hnRNP A1 and TIA1 undergo LLPS and aid the formation of stress granules (SGs) (Murakami et al., 2015; Patel et al., 2015; Lin et al., 2016). Arginine-rich DPRs are able to interact with the LCD of these proteins, disrupting LLPS and altering their biophysical properties (Lee et al., 2016). Therefore, DPRs may contribute to toxicity by upsetting the composition of membrane-less organelles.

Although the precise role of DPR toxicity in *C9orf72*-associated pathogenesis remains controversial, it is evident that their accumulation has detrimental effects on nucleolar function and RNA metabolism. Specific effects of DPR aggregation on RNA processing are discussed in further detail in the following sections.

## Assessing the Relative Contribution of RNA Foci and DPR Accumulation to *C9orf72* Toxicity

Since the discovery of RNA foci and RAN translation products in cells derived from expansion carriers, the relative contribution of these disease parameters to the overall pathomechanism remains a controversial issue. The main argument against the role of DPRs as a main driver of toxicity comes from neuropathological analysis using human brain tissue, which has revealed that DPR distribution is not spatially correlated to severity of degeneration in ALS or FTD (Mackenzie et al., 2013; Davidson et al., 2014, 2016; Gomez-Deza et al., 2015; Schuldi et al., 2015). In fact, DPR load is lower in vulnerable regions (e.g., motor cortex) and higher in unaffected areas (e.g., cerebellum) (Mackenzie et al., 2013), suggestive of a possible neuroprotective role for insoluble DPR aggregates. On the other hand, extensive quantification of RNA foci in the frontal cortex and cerebellum of *C9orf72* expansion carriers revealed limited association between clinic-pathological phenotypes (DeJesus-Hernandez et al., 2017), suggesting that neither RAN translation nor RNA foci can in isolation account for the phenotypic heterogeneity among expansion carriers. Even together, their presence is insufficient to cause disease, as evidenced by a patient with 30× repeats exhibiting both RNA foci and DPRs in the absence of TDP-43 pathology or clinical symptoms (Gami et al., 2015). This suggests that longer repeat lengths overcome a pathological threshold, enabling these initial molecular abnormalities to trigger full-blown disease.

In order to elucidate the relative contributions of RNA toxicity and RAN translation, several experimental strategies employing the use of randomized codon constructs and G_4_C_2_ repeats containing stop codon interruptions have been developed to determine the effects of RNA or DPRs in isolation. The results of these studies are summarized in Table 1. Taken together, these studies suggest that G_4_C_2_ toxicity is mediated through the expression of arginine-rich DPRs, particularly poly-PR, which induce nucleolar stress. However, the fact that we cannot conclusively prove that RNA or DPRs alone act as a main driver of *C9orf72* toxicity indicates that it is a combination of the two, perhaps in parallel with other disease factors, which is ultimately responsible for disease.

**Table 1:** Assessing the relative contributions of RNA and dipeptide repeat protein (DPR) toxicity in *C9orf72* model organisms.

Experimental strategy	Effect in model system	References
Compared “pure repeats” (produced RNA foci and DPRs) with “RNA-only repeats” containing stop-codon interruptions (produced only RNA foci) in *Drosophila*	Eye degeneration occurs only when pure repeats are expressed. Pure repeats have no effect.	Mizielinska et al. (2014)
“Protein-only” constructs employing alternative codons to generate DPRs in *Drosophila*	Arginine-rich DPRs cause eye degeneration and lethality.	Mizielinska et al. (2014)
Alternative codons to generate DPRs expressed in cortical and motor neurons	PR_50_ expression decreased cortical neuron survival. Both PR_50_ and GR_50_ toxic in motor neurons.	Wen et al. (2014)
Constructs containing intronic GGGGCC repeats (which do not initiate RAN translation) expressed in cortical, motor and primary cortical neurons	Increased death of cortical and motor neurons expressing 42× repeats compared to 21× repeats and controls.	Wen et al. (2014); Zhang et al. (2014)
Synthetic GR_20_ and PR_20_ peptides produced using a peptide synthesizer applied to U2OS cells and human astrocytes	PR_20_ and GR_20_ bind nucleoli and kill U2OS cells. Disrupt splicing and ribosomal RNA biogenesis in astrocytes.	Kwon et al. (2014)
GFP-GA_50_ construct consisting of G4C2 repeats with stop codon interruptions expressed in primary neurons	GA_50_ expression stimulates caspase-3 activation, ER stress and decreased proteasome activity	Zhang et al. (2014)
Alternative codons to express poly-GA, poly-GR and poly-GP in *Drosophila*	GR_50_ causes eye degeneration and death. GA and GP expression has no effect.	Freibaum et al. (2015)
Alternative codons to express poly-GR, poly-PR, poly-GA, poly-GP and poly-PA in *Drosophila*	GR_50_ and PR_50_ causes severe eye degeneration. GA_50_, GP_50_ and PA_50_ has no effect.	Lee et al. (2016)
G4C2 repeats flanked by *C9orf72* intronic and exonic sequences which form RNA foci but low levels of DPRs compared to G4C2 repeats with a poly(A) tail producing high levels of RAN translation expressed in *Drosophila*	Intronic construct causes modest toxicity whereas poly(A) construct produces severe eye degeneration.	Tran et al. (2015)
Alternative codons to express poly-GR, poly-PR, poly-GA, poly-GP and poly-PA in *S. cerevisiae*	PR_50_ expression is sufficient to disrupt nuclear pore trafficking	Jovičić et al. (2015)

## Processing of the Expanded *C9orf72* Allele Transcript

**Figure 3:**
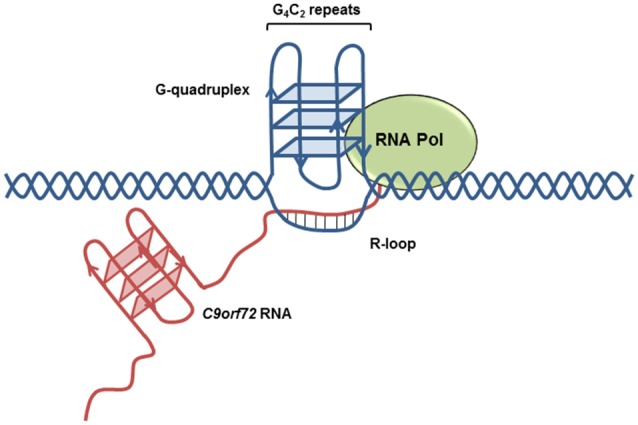
*C9orf72* transcript secondary structures. Highly stable G-quadruplex secondary structures form on the expanded (GGGGCC)_n_ transcript, increasing the likelihood of R-loop formation.

Owing to the high guanine content of the sense transcript, the G_4_C_2_ repeat expansion forms highly stable G-quadruplex structures (Figure 3). G-quadruplexes consist of stacks of planar tetramers in which four guanines interact through Hoogsteen hydrogen bonds. Furthermore, the antisense transcript adopts i-motif secondary structure, consisting of two parallel duplexes held by hemi-protonated C^+^-C pairs, under near physiological conditions (Kovanda et al., 2015). Highly sensitive gene expression profiling identified increased levels of potentially truncated transcripts compared to controls in the frontal cortex, an effect most prominent among patients diagnosed with FTD (van Blitterswijk et al., 2015). Furthermore, a substantial increase in the number of sense and antisense transcripts containing intron 1 (the location of expansion) has been reported among *C9orf72* expansion carriers relative to controls in both cerebellum (Mori et al., 2013b; Niblock et al., 2016) and patient-derived iPSNs (Sareen et al., 2013). This suggests that the presence of the repeat expansion results in preferential use of exon 1a, resulting in a transcriptional bias towards the expanded transcript (Sareen et al., 2013). It is of interest to note that levels of intron 1 containing transcripts are significantly higher in the frontal cortex of FTD patients compared to individuals with ALS (Mori et al., 2013b; van Blitterswijk et al., 2015).

Analysis of *C9orf72* intron 1 splicing demonstrated that a proportion of polyadenylated *C9orf72* transcripts retained intron 1. Intron retention was significantly increased in the frontal cortex from *C9orf72* expansion carriers compared to controls. This effect was especially pronounced in a case homozygous for the *C9orf72* repeat expansion (Niblock et al., 2016). These findings could reflect the aforementioned transcriptional bias towards exon 1a, thereby increasing the total number of transcripts containing the expanded sequence. Of note, a relationship has been established between high intronic GC content and splicing abnormalities (Wong et al., 2013). Furthermore, reduced rates of transcription across the intron owing to complex secondary structure may contribute to perturbed splicing of intron 1.

Taken together, these results suggest that the presence of the expanded G_4_C_2_ sequence and the formation of complex secondary structure results in numerous transcriptional defects, including abortive transcription, increased use of exon 1a and ineffective splicing of the repeat-containing intron.

## Global Splicing Alteration

*C9orf72* RNA foci sequester several members of the hnRNP family of splicing factors (Lee et al., 2013; Mori et al., 2013a; Sareen et al., 2013; Cooper-Knock et al., 2014), resulting in altered splicing patterns of their RNA targets. The production of certain alternatively spliced mRNAs depends on the relative abundance of antagonistically acting splicing factors, highlighting the potential implications of hnRNP sequestration within G_4_C_2_ foci. Multiple lines of evidence point towards perturbations in constitutive and alternative splicing events in *C9orf72* expansion carriers. Gene expression profiling has revealed an increased occurrence of splicing errors in cell lines derived from c9ALS patients, an effect which was most evident among patients with faster disease progression (Cooper-Knock et al., 2015a). Transcriptome analysis of differentially expressed genes between c9ALS and control groups revealed an enrichment of upregulated transcripts involved in RNA splicing (Cooper-Knock et al., 2015a), consistent with a compensatory mechanism for RBP sequestration by RNA foci. Many of the differentially expressed genes have also been independently identified as candidate G_4_C_2_ binding proteins, including hnRNP A3 and hnRNP H (Lee et al., 2013; Mori et al., 2013a). These results suggest that the increased splicing error rate in expansion carriers is a consequence of RBP sequestration into foci, which in turn contributes to disease progression and severity. Consistent with this hypothesis, analysis of abundant RBP motifs among transcripts exhibiting splicing abnormalities revealed an enrichment for binding motifs for hnRNP H (Prudencio et al., 2015). Furthermore, an additional transcriptome analysis reported a three-fold increase in the number of splicing events in c9ALS cases compared to sporadic ALS in both cerebellum and frontal cortex (Prudencio et al., 2015). Intron retention events embodied a significant proportion of such splicing events, revealing a staggering 40-fold increase among c9ALS compared to sporadic ALS within the frontal cortex. Such differences in the extent of intron retention in c9ALS may contribute to the reported increase in nuclear accumulation of mRNA transcripts in *C9orf72*-ALS models (Freibaum et al., 2015; Jovičić et al., 2015; Rossi et al., 2015).

Splicing abnormalities also arise from pathogenic events other than RBP sequestration into foci, such as DPR toxicity. Exposure of cultured cells to arginine-rich DPRs results in their localization to the nucleus where they bind nucleoli, resulting in disruptions in ribosomal RNA biogenesis (Kwon et al., 2014). Furthermore, PR_20_ exposure produced altered splicing patterns of specific mRNA transcripts, including exon skipping and intron retention events. PR_20_ expression caused exon 2 skipping of the mRNA encoding Ran GTPase, producing a protein of reduced length. Ran GTPase functions as a regulator of protein nucleocytoplasmic transport, which has been implicated in *C9orf72*-mediated toxicity and is discussed in further detail below. Exposure to PR_20_ also promotes intron retention within the *GADD45* mRNA, altering the open reading frame and thereby encoding an inactive version of the GADD45 protein (Kwon et al., 2014). Such findings suggest a possible contribution of DPR proteins to the alternative splicing abnormalities documented in c9ALS. Indeed, impaired ribosomal RNA biogenesis and RNA granule formation due to DPR expression suggest that DPRs may perturb RNA processing on a global level (Kwon et al., 2014; Tao et al., 2015).

Thus, TDP-43 loss-of-function, RBP sequestration and the production of DPR species converge to disrupt splicing patterns of mRNAs in various cellular pathways. Perturbations in numerous signaling pathways may explain why so many processes, from autophagy to the stress response, are impaired in *C9orf72*-disease.

## Global Changes in Polyadenylation Site Selection

In addition to alterations in alternative splicing, variations in polyadenylation site (PAS) selection of a large number of transcripts have been identified in the cerebellum of individuals with *C9orf72*-ALS. Analogous to alternative splicing, alternative PAS selection contributes to the complexity of the human transcriptome and has been detected in over 50% of human genes (Tian et al., 2005). Alternative polyadenylation generates mRNA isoforms which vary in their 3′-untranslated regions (3′-UTRs), thus determining the stability, localization and translational efficiency. c9ALS patients show increased use of upstream PASs compared to downstream PASs in the cerebellum compared to sporadic ALS patients (Prudencio et al., 2015). Transcripts exhibiting shifts in PAS selection are enriched for functions in RNA processing, including transcripts encoding exosomal proteins, components of the RISC complex and splicing factors, such as *TARBP2, ATXN2 and EXOSC7* (Prudencio et al., 2015). 3′-UTRs frequently harbor miRNA and RBP binding sites, therefore the selection of upstream PASs, which reduce the length of the 3′-UTR, are likely to result in the loss of such binding sites. mRNA transcripts with shorter 3′-UTRs have an increased stability, resulting in higher protein expression and thereby generating distortions in downstream pathways (Matoulkova et al., 2012). Altered 3′-UTR length may also affect the stability, localization and transport of the mRNA. In addition to its role in alternative splicing, hnRNP H has also been implicated in alternative PAS selection (Chou et al., 1999), suggesting a role for RBP sequestration in disrupted alternative polyadenylation events in *C9orf72*-mediated toxicity.

## Nucleocytoplasmic Transport

**Figure 4:**
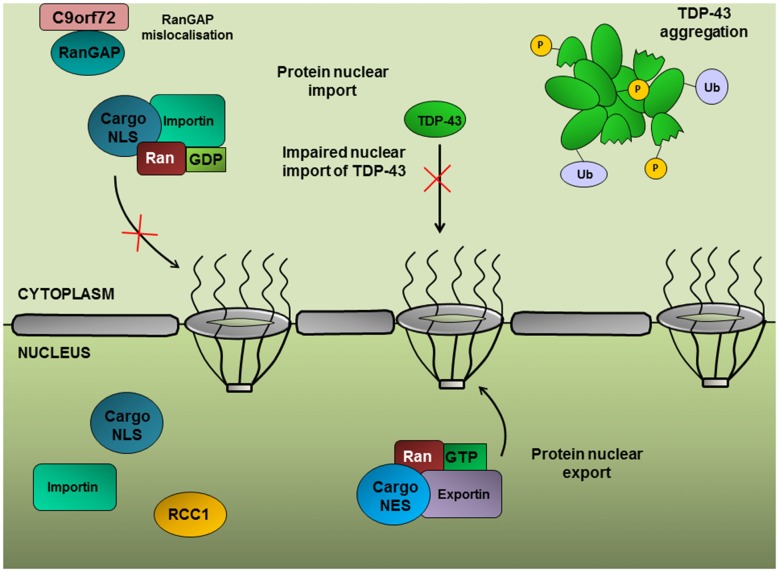
Ran-mediated protein nuclear import. The import of proteins into the nucleus is dependent on a concentration gradient of Ran-GDP:Ran-GTP in the cytoplasmic and nuclear compartments, respectively. RanGAP converts Ran to its GDP-bound form, enabling Ran to shuttle proteins possessing a nuclear localization signal (NLS) into the nucleus. Nuclear import of TDP-43 depends on the Ran-mediated pathway; therefore RanGAP mislocalization could contribute to TDP-43 nuclear depletion.

Mature mRNA transcripts are transported to the cytoplasm from the nucleus through large proteinaceous assemblies embedded in the nuclear envelope, termed NPCs. The mRNA transcript, together with proteins involved in pre-mRNA processing, exist as a large mRNP which interacts with the NPC through nuclear transport receptors. RNA processing reactions generate signals in the form of proteins docked onto the mature mRNA which interact with the nuclear export machinery, ensuring that only fully matured mRNAs are transported to the cytoplasm for translation (Brodsky and Silver, 2000). For example, the UAP56 helicase is retained onto the mRNA after splicing and recruits the nuclear export protein, ALYREF, thereby coupling splicing to nuclear export (Luo et al., 2001). Conversely, following translation, nuclear proteins are transported from the cytoplasm to the nucleus. The vast majority of nuclear proteins are transported from the cytoplasm via the Ran-mediated pathway, which is an energy-dependent directional process reliant on the asymmetric distribution of Ran-GTP in the nucleus and Ran-GDP in the cytoplasm. RanGAP functions as a molecular switch, converting Ran-GTP to Ran-GDP, enabling Ran to shuttle proteins into the cytoplasm and maintaining the gradient of cytoplasmic Ran-GDP (Figure 4). Ran-mediated nucleocytoplasmic trafficking is facilitated by the karyopherin protein family (consisting of importins and exportins which mediate nuclear import and export, respectively) which form a complex with Ran, cargo proteins and components of the NPC.

A genetic screen in *Drosophila* expressing (G_4_C_2_)_30_ repeats identified RanGAP as a potent suppressor of the rough eye phenotype (Zhang et al., 2015), suggesting that upregulation of protein nuclear import can alleviate *C9orf72*-associated toxicity. Furthermore, RanGAP was shown to physically interact with G_4_C_2_ RNA repeats and exhibited mislocalization from the nuclear periphery into large perinuclear aggregates in *Drosophila* expressing (G_4_C_2_)_30_, in patient-derived iPSNs and in the motor cortex from c9ALS patients. A significant correlation between the nuclear to cytoplasmic ratios of Ran and TDP-43 has been established (Ward et al., 2014; Zhang et al., 2015), implying a role for Ran in the nuclear import of TDP-43. Indeed, siRNA knockdown of Ran accessory proteins, such as importin-β1, which function in the nuclear import of proteins with a nuclear localization signal (NLS), results in the cytoplasmic accumulation of TDP-43 (Nishimura et al., 2010). Furthermore, TDP-43 regulates Ran expression through interaction with the 3′-UTR of *Ran* mRNA and TDP-43 inhibition leads to a decrease in *Ran* mRNA and protein expression (Sephton et al., 2011; Ward et al., 2014).

Xiao et al. (2015) reported a direct interaction between the C9orf72 protein with Ran and importin-β1, implicating a potential role for the C9orf72 protein in nucleocytoplasmic trafficking. This group demonstrated redistribution of the C9orf72 short isoform (isoform B) from the nuclear membrane in healthy neurons to the plasma membrane in neurons from c9ALS patients. Loss of C9orf72 isoform B, Ran and importin-β1 from the nuclear membrane correlated with TDP-43 mislocalization and aggregation (Xiao et al., 2015).

Another genetic screen in *Drosophila* expressing (G_4_C_2_)_58_ specifically identified disruptions in the protein and mRNA nuclear export pathways (Freibaum et al., 2015). (G_4_C_2_)_58_ toxicity is exacerbated by disruptions to the CRM1-mediated nuclear export pathway, the pathway responsible for the nuclear export for the majority of proteins and RNA transcripts. Depletion of the nuclear pore components Nup107 or Nup160 which function in RNA nuclear export exacerbates degeneration caused by (G_4_C_2_)_58_. Furthermore, downregulation of *Ref1*, the *Drosophila* ortholog of *ALYREF*, was identified as the strongest suppressor of toxicity (Freibaum et al., 2015). Immunohistochemistry and UV crosslinking have previously demonstrated colocalization to foci and direct binding of ALYREF with both sense and antisense RNA in c9ALS motor neurons (Cooper-Knock et al., 2014, 2015b). Through its interaction with the TREX complex, ALYREF protects the mRNP from exosomal degradation (Chang et al., 2013), therefore the suppressive effect of *Ref1* loss of function results from increased exosomal degradation of nuclear transcripts. A higher proportion of nuclear to cytoplasmic mRNA transcripts were identified in G_4_C_2_-expressing *Drosophila* and c9ALS iPSNs, an effect rescued by *Ref1* knockdown. Moreover, loss of exosomal function was demonstrated to enhance toxicity (Freibaum et al., 2015) and mutations in *EXOSC3* which encodes an exosomal component are responsible for a congenital form of motor neuron disease, pontocerebellar hypoplasia (Wan et al., 2012).

To elucidate the contribution of RNA and DPR toxicity to nucleocytoplasmic defects, a yeast genetic screen was used to generate poly-PR employing codon-optimized constructs without using the G_4_C_2_ repetitive sequence. The strongest suppressors of toxicity were identified as members of the karyopherin family of nuclear import proteins and MTR10, a nuclear import receptor which mediates the import of the serine-arginine (SR) family of splicing factors (Jovičić et al., 2015). It has been previously suggested that DPRs may compete with SR proteins for binding to ribonucleoproteins, as both the SR-domains of splicing factors and synthetic constructs containing 20 GR or PR repeats both bind hnRNP A2 hydrogels (Kwon et al., 2014). Upregulation of MTR10 could alleviate this competitive effect, thereby increasing the influx of SR proteins into the nucleus. This would restore alterations in RNA splicing and processes, such as mRNA nuclear export and nonsense-mediated decay. Consistent with the *Drosophila* genetic screens, the same group also identified components of the NPC and Ran-associated proteins as genetic modifiers of (PR)_50_ toxicity in yeast.

Taken together, the results of these studies suggest that abnormal nucleocytoplasmic transport is a pathological signature of c9ALS/FTD. Disruptions in nucleocytoplasmic transport have also been documented in a mutant SOD1 mouse model of ALS (Zhang et al., 2006), suggestive of an underlying defect common to ALS with different genetic etiologies. Nuclear import and export factors therefore represent a potential therapeutic target for G_4_C_2_ repeat toxicity. ASOs targeting the *C9orf72* transcript have already been shown to normalize Ran and TDP-43 localization in neurons derived from c9ALS iPSCs, presumably through the reduction of G_4_C_2_ RNA foci (Zhang et al., 2015). In addition, small molecules which disrupt the G-quadruplex conformation of G_4_C_2_ RNA rescue nuclear import deficits in the (G_4_C_2_)_30_ *Drosophila* model (Zhang et al., 2015), highlighting the therapeutic amenability of nucleocytoplasmic transport.

## mRNA Localization and Translation

Expanded G_4_C_2_ RNA localizes to neurites where it is subsequently incorporated into active transport granules in *Drosophila* neurons and iPSNs from expansion carriers (Burguete et al., 2015). RNA transport granules are ribonucleoprotein complexes which serve to transport mRNA along microtubules for local protein synthesis. For highly polarized cells, such as neurons, efficient mRNA transport and local translation is crucial for the maintenance of synaptic plasticity. Localization of the G_4_C_2_ repeat RNA to neuronal transport granules is associated with neuritic branching deficits which are modulated by the components of transport granules and translational regulators, FMRP and CPEB3 (Burguete et al., 2015), suggesting that loss of neuritic branches may result from transport of G_4_C_2_ repeat RNA in neurites. Secondary structural conformations, such as G-quadruplexes, appear to promote neuritic localization of G_4_C_2_ repeat RNA and incorporation into RNA transport particles (Subramanian et al., 2011; Burguete et al., 2015), therefore targeting specific structural conformations may promote neuritic branching of neurons in repeat expansion carriers. Of note, binding partners and modifiers of G_4_C_2_ repeat toxicity, including hnRNP A3, hnRNP A2/B1 and Pur-α, are responsible for maintaining transport granule function (Jin et al., 2007; Sofola et al., 2007; Xu et al., 2013).

Impairment in the mRNA nuclear export pathway is predicted to result in reduced translation of mRNA into proteins. Indeed, downregulation of protein translation has been documented in cells expressing (G_4_C_2_)_31_. An RNA pulldown assay using (G_4_C_2_)_31_ revealed binding proteins enriched for functions in translational control, such as EF1α, eIF2α, eIF2β and Pur-α, indicating that protein sequestration by the repeat expansion may also contribute to impaired translation (Rossi et al., 2015). Expression of the G_4_C_2_ sequence in cultured mammalian cells initiates a stress response, characterized by the formation of SGs and a subsequent reduction in the rate of global translation (Rossi et al., 2015). SGs serve to protect the cell during stress, transiently storing mRNAs encoding housekeeping genes and prioritizing the translation of stress-response proteins, such as heat shock proteins and chaperones. SGs are composed of RBPs and poly(A)^+^ mRNAs, which are either transiently stored to resume translation upon alleviation of the stress response or are degraded in processing bodies (P-bodies; Bentmann et al., 2013). Persistent SG formation results in a chronic stress response and prolonged translational repression. Increasing lines of evidence suggest that SG-associated translational repression may play a role in c9ALS/FTD. For example, TDP-43, FUS and Ataxin-2, mutations in which are associated with neurodegenerative disease, all localize to SGs upon cellular stress and may function to regulate SG assembly (Wolozin, 2012; Monahan et al., 2016). Furthermore, genes involved in SG formation are potent modifiers of TDP-43 toxicity in yeast and *Drosophila* (Kim et al., 2014). TDP-43 expression promotes eIF2α phosphorylation, indicative of SG formation and translational repression. Inhibiting SG formation using a small molecule inhibitor of eIF2α alleviates TDP-43 toxicity (Kim et al., 2014). eIF2α is crucial for translation initiation, facilitating the interaction between the ribosome and Met-tRNA. Phosphorylation of eIF2α results in its inactivation, inhibiting global protein synthesis. SG formation can be stimulated by limited availability of eIF2α (Mokas et al., 2009), therefore eIF2α sequestration into RNA foci (Rossi et al., 2015) has the potential to initiate SG formation and translational inhibition. Arginine-rich DPR species impede protein translation in a dose-dependent manner through a mechanism independent of eIF2α phosphorylation (Kanekura et al., 2016). Immunoprecipitation analysis revealed that poly-(PR)_20_ interacts with ribosomal proteins and translation initiation and elongation factors. Furthermore, both poly-(PR)_20_ and poly-(GR)_20_ were shown to aggregate in the presence of mRNA transcripts, obstructing access of the eIF4E and eIF4G translation initiation factors and disrupting proteins translation (Kanekura et al., 2016).

Consistent with reduced nuclear export, nuclear accumulation of poly(A)^+^ mRNA transcripts was detected in (G_4_C_2_)_31_-expressing cells and was strongly associated with relocalization of cytoplasmic poly(A)-binding protein (PABPc) to the nucleus. PABPc directly binds G_4_C_2_ RNA and accumulates into RNA foci (Rossi et al., 2015). Of note, targeting PABPc to the nucleus is sufficient to cause nuclear accumulation of mRNAs (Kumar and Glaunsinger, 2010), therefore nuclear sequestration of PABPc by G_4_C_2_ foci may represent a potential link between RNA toxicity and nuclear retention of mRNAs. Furthermore, PABPc plays a key role in regulating mRNA translation by facilitating the interaction between translation initiation factors and the mRNA 5′ cap, enhancing ribosomal recruitment. PABPc nuclear sequestration may reduce global translational efficiency of the depleted pool of mRNAs present in the cytoplasm. Thus, sequestration of translational regulators by RNA foci may result in the nuclear retention of mRNAs and a concomitant reduction of translation in the cytoplasm.

## Impaired RNA Processing Is Associated with R-Loop Formation

The occurrence of the G-quadruplex or i-motif structures from the sense or antisense transcript, respectively, displaces the complementary DNA strand, favoring the formation of R-loop secondary structures. R-loops form during transcription when the nascent RNA molecule interacts with displaced DNA, resulting in the formation of a three stranded nucleic acid structure consisting of an RNA:DNA hybrid and single stranded DNA (ssDNA) (Figure 3), taking advantage of the increased thermodynamic stability of RNA:DNA hybrids compared to the DNA duplex. Conversely, perturbations in RNA processing can result in increased formation of R-loop structure (Huertas and Aguilera, 2003; Wahba et al., 2011). R-loops may reduce transcriptional efficiency resulting in the formation of aborted transcripts. An *in vitro* transcription assay using G_4_C_2_ repeats that form G-quadruplex structures demonstrated a repeat-length dependent accumulation of transcripts truncated within the repeat expansion region. RNase H treatment, which specifically digests the RNA transcript within DNA-RNA duplexes and resolves R-loop structures, reduces abortive transcripts (Haeusler et al., 2014). Of note, mutations in senataxin, an RNA helicase which resolves R-loop structures, are causative of ALS4, a juvenile form of ALS (Chen et al., 2004). Depletion of the splicing factor ASF/SF2, a protein shown to bind G_4_C_2_ repeat sequences (Reddy et al., 2013; Rossi et al., 2015), results in a concomitant increase in R-loop formation (Li and Manley, 2005). Furthermore, R-loops are a known source of DNA damage (Huertas and Aguilera, 2003) and are likely to contribute to the upregulation of DNA damage markers identified in ALS motor neurons (Farg et al., 2017).

In addition to *C9orf72*-linked disease R-loop formation has been characterized in several microsatellite disorders, such as Fragile X Syndrome (FXS) and Friedrich’s Ataxia (FRDA) (Groh et al., 2014). R-loops were found to be enriched over GAA repeats associated with FRDA and the abundance of R-loops correlated with repeat expansion length. Furthermore, increased formation of R-loops resulted in upregulation of repressive chromatin, promoting transcriptional silencing of the *FXN* gene (Groh et al., 2014). Therefore, R-loop formation may contribute to some of the processes characteristic of repeat expansion disorders, including repeat instability, antisense transcription and impaired transcription. Incubation of transcriptionally induced R-loops with cell lysates stimulated variations in repeat length within the DNA template (Reddy et al., 2014), suggesting that R-loop formation can have direct consequences on repeat instability. It remains unclear how R-loops promote repeat instability, however it has been proposed that R-loop structures favor a non-B-DNA structure of the ssDNA, resulting in the recruitment of various DNA repair factors which mediate error-prone DNA repair synthesis (Lin et al., 2010). R-loops also have the ability to induce transcriptional pausing or stalling, perhaps by forming a structural block or by triggering mechanisms of transcriptional repression, such as histone methylation (Groh et al., 2014; Skourti-Stathaki et al., 2014). Additionally, R-loops may induce expression of the antisense transcript (Skourti-Stathaki et al., 2014). The fact that R-loop formation is associated with so many features of *C9orf72* toxicity makes it an imperative area of future research and a potential attractive therapeutic target applicable to a range of microsatellite repeat disorders. Small molecules able to intercalate within G-quadruplex or hairpin structures have demonstrated promising suppression of R-loops arising from trinucleotide expansions (Colak et al., 2014).

## Concluding Remarks

It is becoming clear that impaired RNA processing is a central feature of c9ALS/FTD pathogenesis which is mediated via several interconnected mechanisms, including sequestration of RBPs, RAN translation and TDP-43 mislocalization. It is likely that these mechanisms all contribute to RNA processing abnormalities to different extents, generating numerous small defects at the level of RNA metabolism. Initially the cell may be able to compensate for these defects, however over time these abnormalities may accumulate beyond a certain threshold at which point the aging cell loses viability. The idea of c9ALS/FTD as a disorder mediated by an accumulation of several minor disruptions, rather than a few isolated RNA misprocessing events as has been described for myotonic dystrophy type 1 (Timchenko et al., 2001; Jiang et al., 2004), may in part account for the extreme variability in clinical phenotypes exhibited by *C9orf72* expansion carriers. Given the importance of RNA processing in cellular function, RNA metabolism can be considered an essential target for therapeutic intervention to improve the lives of those living not only with c9ALS/FTD but for a range of neurodegenerative disorders.

## Author Contributions

HVB wrote the manuscript. MN, Y-BL and CES reviewed the manuscript. J-MG reviewed and edited the manuscript.

## Conflict of Interest Statement

The authors declare that the research was conducted in the absence of any commercial or financial relationships that could be construed as a potential conflict of interest.
